# Targeting FHL2-E-cadherin axis by miR-340-5p attenuates colon cancer cell migration and invasion

**DOI:** 10.3892/ol.2021.12898

**Published:** 2021-07-03

**Authors:** Anwar Algaber, Raed Madhi, Avin Hawez, Carl-Fredrik Rönnow, Milladur Rahman

**Affiliations:** 1 Department of Clinical Sciences, Malmö, Section for Surgery, Lund University, 214 28 Malmö, Sweden; 2 Department of Biology, College of Science, University of Misan, Maysan 62001, Iraq

**Keywords:** colon cancer, four and a half LIM domains protein 2, microRNAs, cell migration, metastasis

## Abstract

Convincing data has suggested that four and a half LIM domain 2 protein (FHL2) serves a key function in cancer cell metastasis and that microRNA (miR)-340-5p can regulate cancer cell migration. The current study hypothesized that targeting FHL2 expression by miR-340-5p in colon cancer may attenuate colon cancer cell migration and invasion. FHL2 expression was therefore assessed in colon cancer microarray datasets using Qlucore omics explorer as well as in HT-29 and AZ-97 colon cancer cell lines via reverse transcription-quantitative PCR (RT-qPCR). Colon cancer cell migration and invasion were evaluated in the presence of miR-340-5p mimic, mimic control or mimic with a target site blocker. Confocal microscopy and RT-qPCR were subsequently performed to assess FHL2, E-cadherin (E-cad) protein and mRNA expression in colon cancer cells. Microarray dataset analysis revealed that FHL2 expression was lower in primary colon cancer cells compared with normal colonic mucosa. It was revealed that the expression of miR-340-5p and FHL2 were inversely related in serum-grown and low-serum conditions in HT-29 and AZ-97 cells. Short-time serum exposure to low-serum grown cells induced FHL2 expression. Transfection of HT-29 cells with miR-340-5p mimic not only decreased serum-induced expression of FHL2 but also decreased cancer cell migration and invasion. Bioinformatics analysis revealed that FHL2 mRNA had one putative binding site for miR-340-5p at the 3-untranslated region. Blocking of the target site using a specific blocker reverted miR-340-5p mimic-induced inhibition of FHL2 expression and cancer cell migration and invasion. Confocal microscopy confirmed that the reduction of FHL2 expression by miR-340-5p mimic also reversed serum-induced E-cad disruption and that the target site blocker abrogated the effect of miR-340-5p. The current results suggested that miR-340-5p could be used to antagonize colon cancer cell metastasis by targeting the FHL2-E-cad axis.

## Introduction

Colon cancer (colon and rectal) is the second most frequent cause of cancer-related death in the world (1). The overall prognosis of colon cancer has improved over the past decades owing better surgical techniques as well as improved neo-adjuvant and adjuvant treatment. Despite this, the prognosis is still poor when distant metastasis are present (Stage IV colon cancer) with a 5 year relative survival rate of 12% (2). Approximately 20% of colon cancer patients have metastatic disease at diagnosis and more than 30% of patients with colon cancer develop metastasis over time (3). The process of metastasis is complex and regulated by a wide range of cellular and molecular changes including alterations of cytoskeleton protein and microRNA (miRNA). Several studies have shown that stress conditions such as hypoxia, lack of nutrients and pH alterations can induce up-regulation of pro-metastatic genes (4–6). Alterations of these genes can subsequently induce cancer cell migration and invasion, further aggravating the disease.

Four and a half LIM domain 2 protein (FHL2) is a multifunctional oncoprotein involved in cancer progression and metastasis (7,8). FHL2 is known to modulate intracellular signaling pathways by post-translational modifications of proteins or by altering gene expression via interactions with transcription factors (9). The expression patterns of FHL2 are different in different cancer types and FHL2 has been shown to be down-regulated in some cancer forms such as prostate cancer (10) and acute myeloid leukemia (11) whilst up-regulated in others such as breast cancer (12), ovarian cancer (13), cervical cancer (14) and colon cancer (8). In one recent publication FHL2 was shown to regulate ovarian cancer cell metastasis via Wnt/β-catenin signaling (7). In addition, FHL2 has been demonstrated to promote epithelial-mesenchymal transition (EMT) in colon cancer cells (8) which is known to be associated with cancer progression and metastasis (15). Hence, FHL2 plays an important role in cancer cell invasion and migration through multiple different mechanisms.

E-cadherin (E-cad) is an important epithelial cell adhesion molecule and loss of its function is associated with epithelial cancer cell migration and invasion (16). E-cad suppresses cancer cell metastasis by preventing the dislodging of cancer cells from the primary tumor and thereby reduce cancer cell dissemination (17). Thus, cancer cell migration and E-cad levels are inversely related and invasion as well as metastasis of cancer cells have been reported following loss of E-cad (18,19). In contrast, E-cad has also been reported to potentiate metastasis since it enhances cancer cell survival (20). In addition, down-regulation of E-cad by FHL2 is associated with EMT in colon cancer (21). Thus, finding a way of controlling both FHL2 and E-cad could potentially be a therapeutic approach in antagonizing colon cancer metastasis.

miRNAs are 21–22 base pair long non-coding RNAs that post-transcriptionally down-regulate mRNA by targeted degradation or blocking translation initiation (22). It is estimated that more than 60% of coding genes in humans have miRNA binding sites in their 3′-untranslated region (3′-UTR) (23). Accumulating data suggest that a single miRNA may target multiple transcripts and a single transcript can be regulated by multiple miRNAs within a cell type (24,25). Some miRNAs are also known to regulate cell adhesion process and thereby regulate cancer invasion and metastasis (26). Interestingly, some cancer-associated miRNAs can act as both oncomir and tumor suppressive miRNA, dependent on the context and type of cancer (27). For instance, up-regulation of miR-340-5p has been shown to promote cancer cell proliferation and progression in thyroid cancer (28) and gastric cancer (29). In contrast, several studies have shown that miR-340-5p is down-regulated in glioblastoma multiforme (30) and breast cancer (31). In addition, one study suggests that lower expression of miR-340-5p is associated with higher levels of FHL2 in ovarian cancer (7). However, the interaction between miR-340-5p and FHL2 and their role in regulating colon cancer cell migration and invasion has not yet been investigated.

Based on the considerations above, we hypothesized that miR-340-5p might regulate colon cancer cell migration and invasion via targeting FHL2-E-cad axis. For this purpose, we used metastatic human colon cancer cell lines to evaluate miR-340-5p-mediated suppression of colon cancer cells migration and invasion.

## Materials and methods

### 

#### Microarray database analysis

Four human colon cancer-related gene microarray datasets (GDS4382, GSE115313, GDS4393 and GDS4516) were downloaded from the Gene Expression Omnibus (GEO) database of NCBI (National Center for Biotechnology Information). GDS4382 and GSE115313 were used to compare FHL2 expression between normal colon mucosa and colon cancer tissue. GDS4393 and GDS4516 were used to compare FHL2 expression between primary colon cancer, metastatic cancer and metastatic recurrence samples. GDS4382 dataset contains 17 colon cancer tumors and 17 adjacent non-cancerous tissues. GSE115313 data set contains 42 paired tumor and normal colon mucosa samples from a cohort study of 42 colon cancer patients. GDS4393 dataset contains 33 primary and 21 metastatic lesions from patients with unresectable colon cancer. GDS4516 dataset contains 66 non metastasized tumors, 20 metastasized tumors and 18 metastatic recurrence tumors of colon cancer patients. GDS4382, GDS4393 and GDS4516 datasets were from GPL570: [HG-U133_Plus_2] Affymetrix Human Genome U133 Plus 2.0 Array platform. GSE115313 dataset was from GPL16686: [HuGene-2_0-st] Affymetrix Human Gene 2.0 ST Array [transcript (gene) version] platform. Qlucore Omics Explorer version 3.6 (32) was used for gene expression analysis and box plots generation. Tukey's post-hoc test was used for statistical comparison between two groups.

#### Cells and reagents

The human epithelial colorectal cancer cell line HT-29 was obtained from American Type Culture Collection (HTB-38, ATCC). HT-29 cell line was characterized to have metastatic properties (32,33). AZ-97 cell line was isolated from a 76-year-old female patient undergoing surgical resection and established in our laboratory at Skåne University Hospital, Malmö, Sweden (34). Cells were cultured in Dulbecco's modified Eagle's medium (DMEM; Sigma-Aldrich), in the presence of 10% fetal bovine serum (FBS) and antibiotics (100 U/ml penicillin, 100 µg/ml streptomycin) at 37°C and 5% CO_2_. miR-340-5p mimic and mimic-Ctrl were purchased from Life Technologies. TransIT-TKO transfection reagent (Mirus) was used to evaluate the role of miR-340-5p. To study the biological function of miR-340-5p, a target site blocker (TSB) was purchased from Exiqon A/S (Vedbaek). The miRCURY LNA_TSB was designed to specifically compete with the miR-340-5p.

#### Cell transfection

HT-29 and AZ-97 colon cancer cells were cultured to 70–80% confluency. After that cells were serum starved (0.1% serum) overnight. On the next day 1×10^6^ cells were seeded in a 6-well culture plate. Cells were then transfected with miR-340-5p mimic (50 nM) or mimic-Ctrl (50 nM) for 24 h by using Mirus transfection reagent in Opti-MEM reduced serum media according to manufacturer's instructions. After 24 h, cells were harvested and expression of miR-340-5p and FHL2 was analyzed by RT-qPCR. To evaluate the effect of short serum exposure to serum starved cells, transfected cells were cultured in Opti-MEM reduced serum media for 24 h and then exposed to 10% BSA for 30 min. RNA samples were extracted by using TRIzol (Invitrogen, Thermo Fisher Scientific, Inc.) and purified using Direct-zol RNA extraction kit (Zymo Research) according to manufacturer's recommendations. cDNA was synthesized using total RNA (0.4 µg) by Mir-XTM miRNA First-Strand Synthesis Kit. Expression of miR-340-5p, FHL2 and E-cad mRNA were quantified using miR-X™ miRNA RT-qPCR SYBR^®^ kit (Clontech). The PCR primers used were as follows; hsa-miR-340-5p specific sense 5′-GGCTTATAAACGAATCACAGTCATTAAAA-3, FHL2 mRNA sense; 5′-GAAACTCACTGGTGGACAAGC-3′, antisense; 5′-GTGGCAGATGAAGCAGGTCT-3′, E-cad mRNA sense; 5′-ACAGCCCCGCCTTATGATT-3′, antisense; 5′-TCGGAACCGCTTCCTTCA-3′, U6 sense; 5′-GCTTCGGCAGCACATATACTA-3′, U6 antisense; 5′-CGAATTTGCGTGTCATCCTTG-3′, β-actin sense; 5′-AGAGCCTCGCCTTTGCCGATCC-3′, antisense; 5′-CACATGCCGGAGCCGTTGTCG-3. Expression of miR-340-5p relative to U6 snRNA and expression of FHL2 as well as E-cad relative to β-actin were determined using 2^−ΔΔCq^ method.

#### Target site blocking of miR-340-5p

Target Scan prediction tool was used to predict binding site for miR-340-5p at the 3′-UTR of FHL2 mRNA (http://www.targetscan.org/). To evaluate the function of the binding site, 20 nucleotides long target site blocker (TSB) was designed to specifically compete with the mir-340-5p in the 3′-UTR of FHL2 mRNA. The miRCURY LNA_TSB The blocker was synthesized as fully phosphorothiolated Locked Nucleic Acids (LNA) in the DNA sequences to increase their affinity and selectivity for the target. Under serum starved conditions, the target site blocker TSB_FHL2_miR-340-5p; 5′-TTATAAAGTAGTTACAGCCT-3′ were co-transfected with the miR-340-5p mimic (50 nM). FHL2 and E-cad mRNA and proteins levels were quantified using RT-qPCR and confocal imaging, respectively.

#### Chemotaxis and invasion assays

Migration and invasion assays were performed using 24-well cell migration chambers with 8 µm pore size inserts (Corning Coster) as described previously (32). For invasion assay, each chamber was coated with 30 µm of extracellular matrix (ECM) gel (Sigma-Aldrich). HT-29 cancer cells were transfected with either miR-340-5p-mimic (50 nM) alone or mimic-Ctrl (50 nM) alone or miR-340-5p (50 nM) in the presence of TSB or TSB-Ctrl for 24 h in Opti-MEM serum reduced media. Next day, transfected cells were collected and 1×10^6^ cells/ml were loaded into the inserts and DMEM media containing 10% serum was added in the lower chambers and incubated for 24 h at 37°C in 5% CO_2_. Non-migrated cells were removed from the upper surface of the insert using cotton swab and cells on the lower surface of the insert membrane were fixed in ice-cold 100% methanol and stained with 0.5% crystal violet. Cells were counted in five different high power field (HPF). Data are expressed as the mean number of migrated cells per high power field.

#### Confocal microscopy

For immunofluorescence imaging of E-cad and ki67, cancer cells were grown to 60–70% confluency and then cells were transfected with either miR-340-5p mimic (50 nM) alone or mimic-Ctrl (50 nM) alone or miR-340-5p (50 nM) in the presence of TSB or TSB-Ctrl for 24 h in Opti-MEM serum reduced media as on glass coverslips as described above. Next day, cells were exposed to 10% BSA for 30 min. Cells were then fixed with 2% formaldehyde and permeabilized with 0.2% Triton X-100 for 20 min. After fixation and permeabilization, cells are then washed two times with PBS containing 2% fetal bovine serum. Samples were then incubated with primary antibodies: Fluorescein isothiocyanate (FITC) conjugated anti-ki67 antibody (ab206633; Abcam) and rabbit anti-human FHL2 antibody (ab12327; Abcam) in PBS containing 2% BSA overnight. In a separate experiment for E-cad staining, samples were first incubated with rabbit anti-human E-cad (ab40772; Abcam) primary antibody in PBS containing 2% BSA overnight. After washing two times, all samples were incubated with rat anti-rabbit allophycocyanin (APC) conjugated secondary antibody (A-21038; Thermo Fisher Scientific, Inc.) for 1 h. After immunostaining, coverslips were rinsed with PBS twice and then stained with Hoechst 33258 (Thermo Fisher Scientific, Inc.) for 10 min. ProLong Diamond Antifade Mountant (Thermo Fisher Scientific, Inc.) was added on all coverslips before putting on the slides. Confocal z-stakes images were taken using LSM 800 confocal microscope (Carl Zeiss) and orthogonal projection images were created using all slices for a total height of ~10 µm. Images were taken by using a ×63 oil immersion objective (numeric aperture=1.25) and processed later using ZEN2012 (Carl Zeiss) software.

#### Statistical analysis

Statistical analyses for *in vitro* experiments were performed using GraphPad Prism 8 software. For multiple comparisons, we used one-way analysis of variance (ANOVA) followed by the Tukey's post hoc test. For comparison between two groups, we used two-tailed t-test. P<0.05 was considered to indicate a statistically significant difference.

## Results

### 

#### FHL2 expression in colon cancer

**Figure 1.**
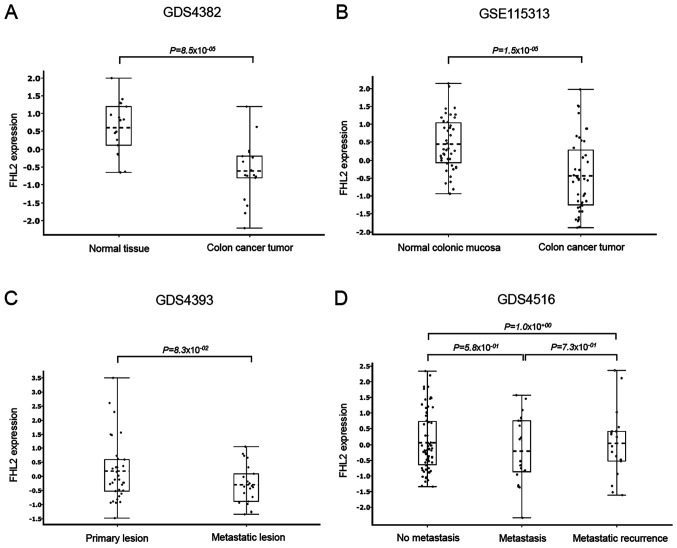
Expression of FHL2 in colon cancer from different microarray datasets. FHL2 expression was compared in normal colon mucosa and colon cancer tissue in (A) GDS4382 and (B) GSE115313 datasets. FHL2 expression was also compared in patients with primary colon cancer, metastatic colon cancer and recurrence metastasis in (C) GDS4393 and (D) GDS4516 datasets. Box plots were created using Qlucore program and represent the mean (25–75 percentile), with whiskers indicating the minimum and maximum levels, and dots represent log_2_ normalized sample expression values. FHL2, four and a half LIM domains protein 2

Knowing that FHL2 expression is different in different tissues, we first compared FHL2 expression between normal colon mucosa and colon cancer in different datasets. Datasets GDS4382 and GSE115313 analysis revealed that colon cancer tissue had significantly lower levels of FHL2 expression than matched normal non-cancerous colonic tissue (Fig. 1A and B). We also compared primary colon cancer samples with metastatic and recurrence metastatic colonic tissues in two different datasets (GDS4393 and GDS4516), it was found that there were no significant differences between primary colon cancer and metastatic colon cancer tissues (Fig. 1C and D). In addition, we also analyzed FHL2 expression among some common colon cancer cell lines and found that FHL2 expression was relatively low in HT-29 and HCC2998 cell lines compared to other cell lines such as CaCo2, DLD-1, HCT116, HCT15, LoVo, SW480 and TC71 (Fig. S1A). We assessed FHL2 expression between HT-29 and AZ-97 in low-serum condition and observed that FHL2 expression is higher in AZ-97 cell line compared to HT-29 cell line (Fig. S1B), suggesting that AZ-97 cell line is similar to CaCo2, DLD-1, HCT116, HCT15, LoVo, SW480 and TC71 cell lines in terms of FHL2 expression. Knowing that FHL2 levels are different in different cancer types, we analyzed a dataset (GSE103512) containing four different cancer samples, our analysis revealed that colon cancer and prostate cancer had similar levels of FHL2 expression while breast cancer and non-small cell lung cancer had significantly lower levels of FHL2 expression than colon cancer and prostate cancer (Fig. S2).

#### miR-340-5p reduces FHL2 expression in colon cancer

**Figure 2.**
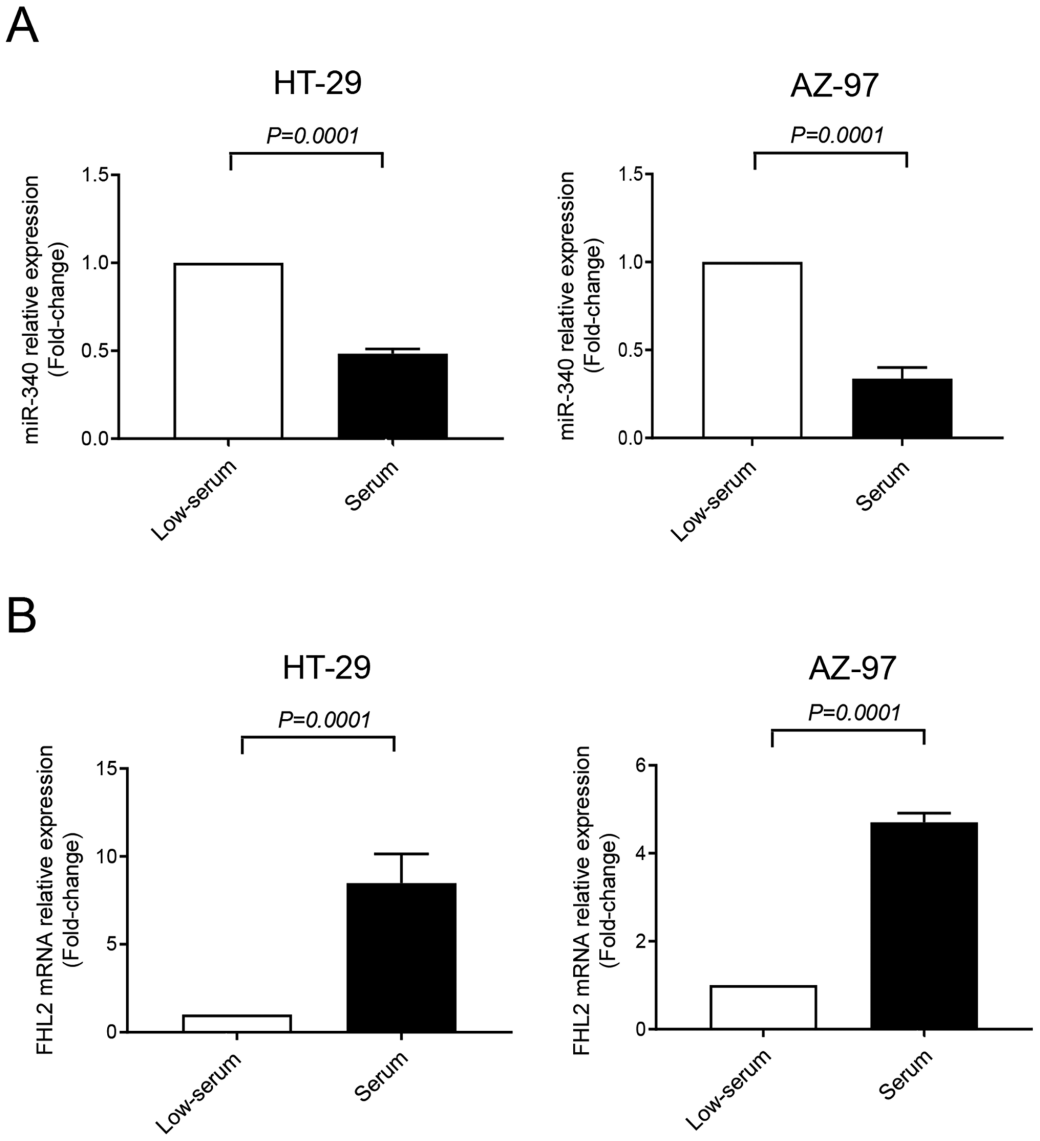
Expression of miR-340-5p and FHL2 in HT-29 and AZ-97 colon cancer cell lines. Expression of (A) miR-340-5p and (B) FHL2 mRNA in low-serum and serum-grown HT-29 and AZ-97 cells. Relative expressions were determined using reverse transcription-quantitative PCR with U6 as a control for miR-340-5p and β-actin as a control for FHL2 mRNA. Relative expressions were determined using the 2^−ΔΔCq^ method. Data are presented as the mean ± SEM (n=4). miR, microRNA; FHL2, four and a half LIM domains protein 2.

**Figure 3.**
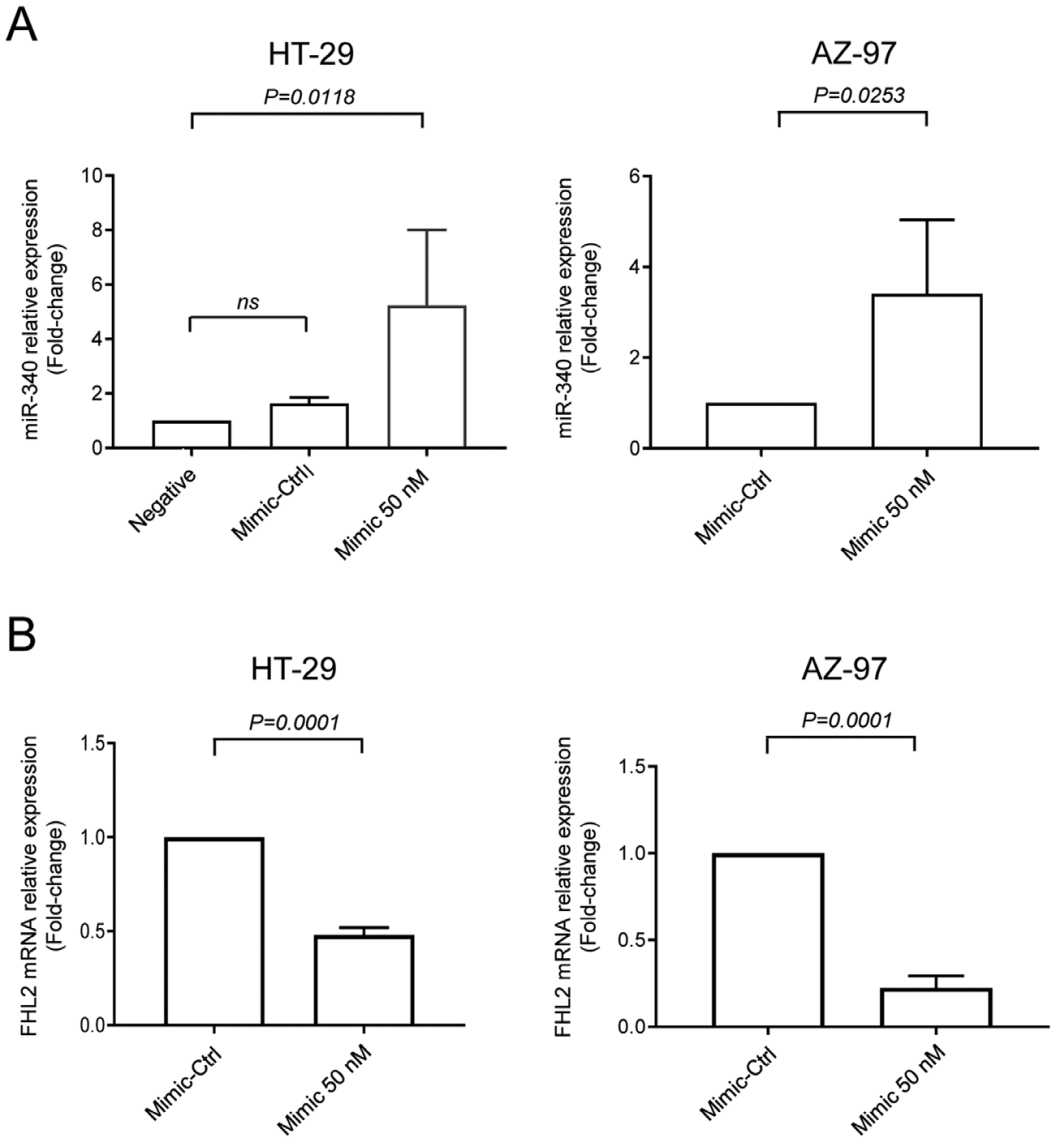
miR-340-5p regulates FHL2 mRNA expression in HT-29 and AZ-97 colon cancer cells. Transfection with 50 nM miR-340-5p mimic (A) upregulated miR-340-5p and (B) downregulated FHL2 mRNA expression in colon cancer cells. Relative expressions were determined by performing reverse transcription-quantitative-PCR where U6 was used as a a control for miR-340-5p and β-actin was used as a control for FHL2 mRNA. Relative expressions were determined using the 2^−ΔΔCq^ method. Data are presented as the mean ± SEM (n=4). miR, microRNA; FHL2, four and a half LIM domains protein 2; Ctrl, control.

Expression of miR-340-5p and FHL2 were assessed in HT-29 and AZ-97 colon cancer cell lines in low-serum (to mimic stress condition of tumor microenvironment) and serum-grown culture conditions by RT-qPCR. It was found that expression of FHL2 mRNA was significantly higher in serum-grown HT-29 and AZ-79 cell lines compared to low-serum condition (Fig. 2B). Furthermore, levels of miR-340-5p were significantly lower in serum-grown cancer cells compared to low-serum cultured cells (Fig. 2A), indicating an inverse relationship between FHL2 mRNA and miR-340-5p in colon cancer cells. Low-serum condition was used for the subsequent experiments of the study. Next, cells were transfected with miR-340-5p mimic or mimic-Ctrl. We found that levels of miR-340-5p were increased when transfected with miR-340-5p mimic (Fig. 3A). Interestingly, transfection of both cell lines with miR-340-5p mimic reduced levels of FHL2 mRNA (Fig. 3B).

#### FHL2 contains functional binding site for miR-340-5p

**Figure 4.**
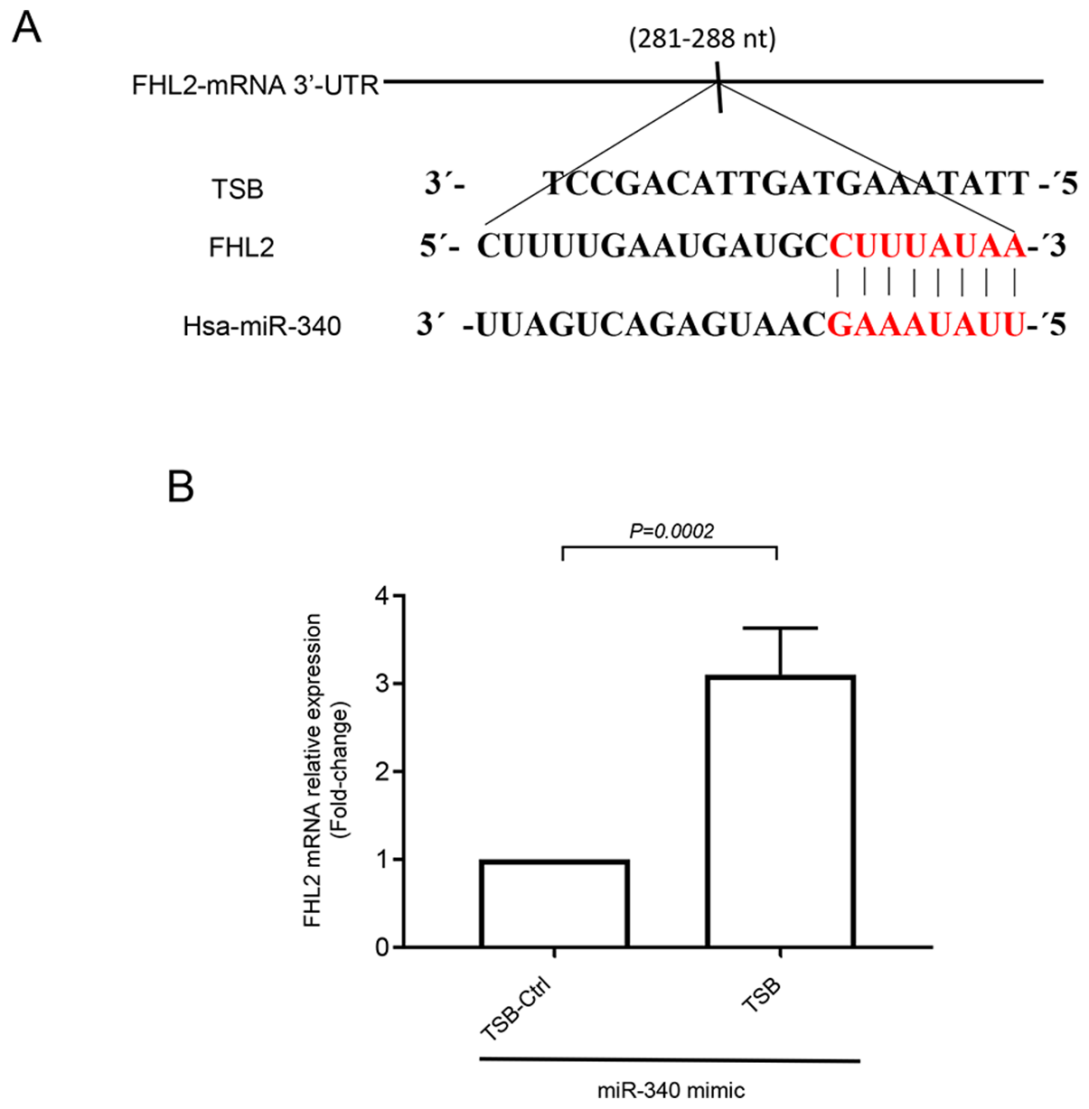
miR-340-5p directly targets FHL2. (A) The predicted target site of miR-340-5p in the 3′-UTR sequence of FHL2 mRNA containing an AAUAUUUC motif is presented. The seeding region of miR-340-5p complementary to UUAUAAAG was then blocked using TSB (red sequence). (B) TSB (50 nM) reversed the inhibitory effect of miR-340-5p (50 nM) on FHL2 mRNA expression in HT-29 colon cancer cells. Data are presented as the mean ± SEM (n=4). miR, microRNA; FHL2, four and a half LIM domains protein 2; TSB, target site blocker; nt, nucleotide; Ctrl, control.

To investigate whether FHL2 oncogene had any binding site for miR-340-5p, we utilized bioinformatics based target site prediction tool (TargetScan). Our analysis revealed that there is one potential binding site for miR-340-5p at 3′-UTR of FHL2 mRNA where it contains complementary sequences of perfect 8′mer base-pair match to the seeding region of miR-340-5p (Fig. 4A). We designed a specific target site blocker (TSB) for FHL2 mRNA. We observed that co-transfection of TSB with miR-340-5p mimic reversed the inhibitory effect of miR-340-5p mimic on FHL2 mRNA expression. In contrast, co-transection with a control TSB had no effect on the level of FHL2 mRNA expression in colon cancer cells (Fig. 4B), confirming that miR-340-5p specifically targets FHL2 mRNA in HT-29 colon cancer cell line.

#### miR-340-5p inhibits FHL2 expression and proliferation of colon cancer cells

**Figure 5.**
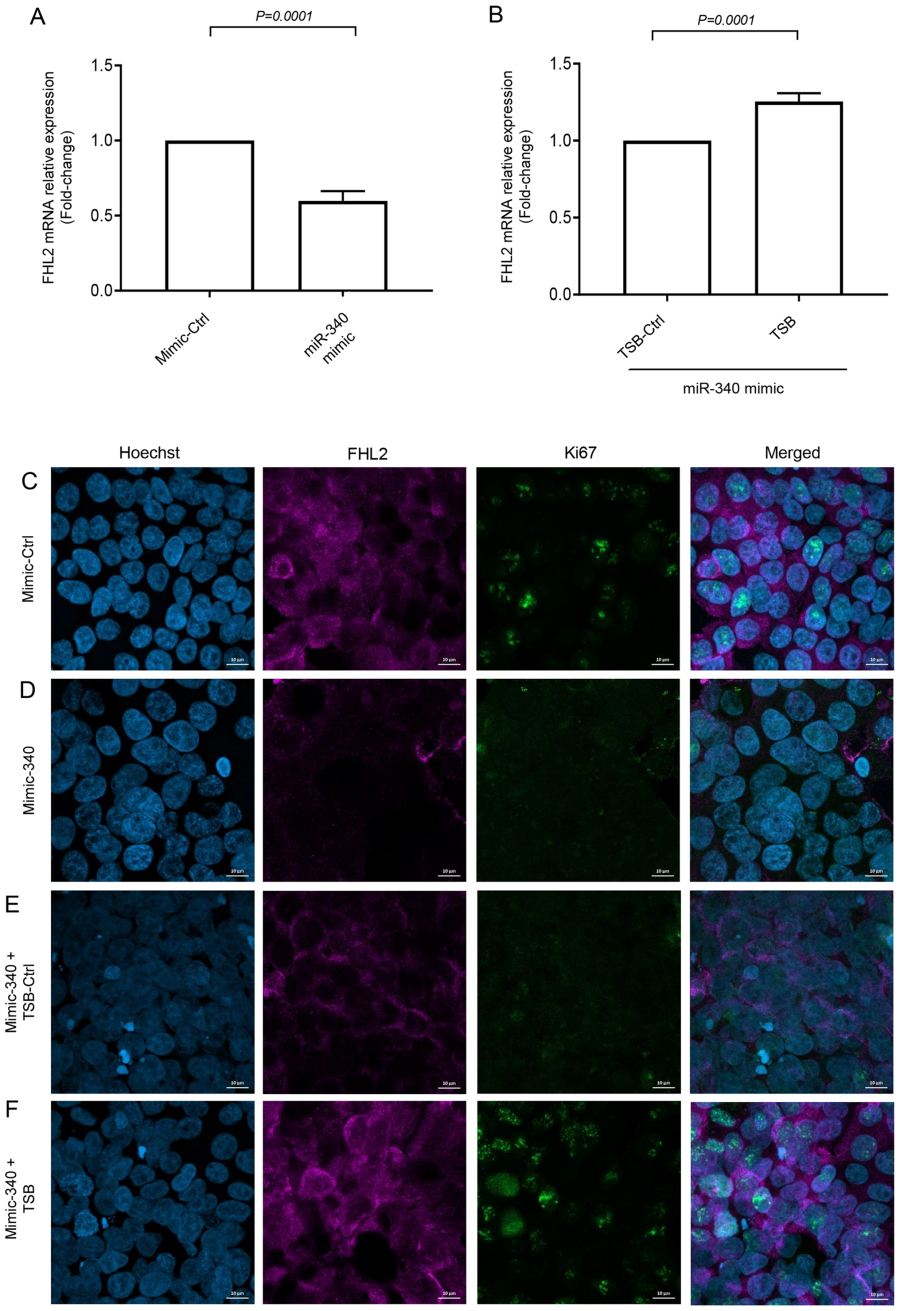
miR-340-5p downregulates FHL2 expression in colon cancer cells. FHL2 expression was evaluated using (A and B) RT-qPCR and (C-F) confocal microscopy (scale bars, 10 µm). Cells were transfected with miR-340-5p mimic, mimic control, TSB control and TSB for 24 h in low-serum conditions. Colon cancer cells were then stimulated using 10% FBS for 30 min. Relative expressions of FHL2 mRNA was quantified using RT-qPCR where β-actin was used as an internal control for FHL2 mRNA. Expressions were determined using 2^−ΔΔCq^ method. Data are presented as the mean ± SEM (n=4). miR, microRNA; FHL2, four and a half LIM domains protein 2; RT-qPCR, reverse transcription-quantitative PCR; miR, microRNA; Ctrl, control.

Short-time (30 min) serum stimulation increased FHL2 mRNA expression and transfection of miR-340-5p mimic was found to reduce the expression of FHL2 mRNA (Fig. 5A). Moreover, co-transfection of miR-340-5p mimic with TSB significantly increased levels of FHL2 mRNA expression (Fig. 5B). Confocal microscopy revealed that serum stimulation increased FHL2 levels in the cytoplasm of the cancer cells (Fig. 5C), and miR-340-5p mimic treatment reduced FHL2 levels (Fig. 5D). Co-transfection of TSB with miR-340-5p mimic reversed the effect of miR-340-5p mimic in serum-stimulated cells but not by the TSB control (Fig. 5E and F). In addition, we found that miR-340-5p mimic reduced cancer cell proliferation in terms of ki67 expression and co-transfection with TSB reversed miR-340-5p mimic-induced inhibition of colon cancer cell proliferation but not by TSB control (Fig. 5C-F).

#### miR-340-5p inhibits colon cancer cell migration and invasion

**Figure 6.**
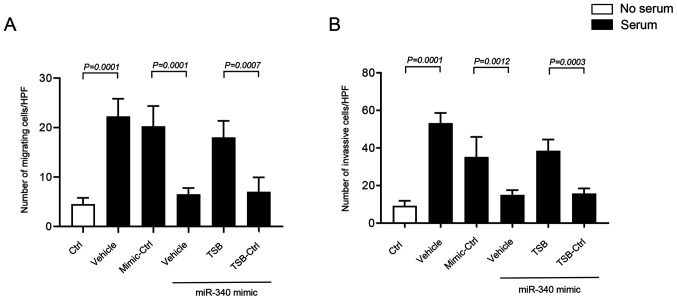
miR-340-5p regulates colon cancer cell migration and invasion. Cells were transfected with miR-340-5p mimic, mimic control, TSB control and TSB for 24 h in low-serum conditions. Serum-stressed transfected cells were then loaded in the upper chamber of the trans-well equipment, with the lower chamber filled with 10% bovine serum albumin. (A) Migration of colon cancer cells toward serum. (B) Invasion of colon cancer cells towards serum. Cells were counted microscopically in five different fields. Data are presented as the mean ± SEM (n=4). miR, microRNA; TSB, target site blocker; Ctrl, control; HPF, high power field.

Transwell migration and invasion assays were performed using 10% FBS as a chemoattractant in the lower chamber of the wells. It was found that miR-340-5p regulates colon cancer cell invasion and migration by targeting FHL2. Serum stimulant significantly increased invasion and migration of HT-29 cells (Fig. 6A, B). Interestingly, transfection of HT-29 cells with miR-340-5p mimic significantly reduced invasion and migration of cancer cells (Figs. 6A and B, and S3, S4). Moreover, it was found that co-transfection of miR-340-5p mimic and TSB, but not with control TSB, had significantly higher invasion and migration of cancer cells (Figs. 6A and B, and S3, S4), suggesting that miR-340-5p inhibits cancer cell migration and invasion by targeting a specific binding site at the 3′-UTR of FHL2 mRNA.

#### miR-340-5p up-regulates E-cadherin expression in colon cancer cells

**Figure 7.**
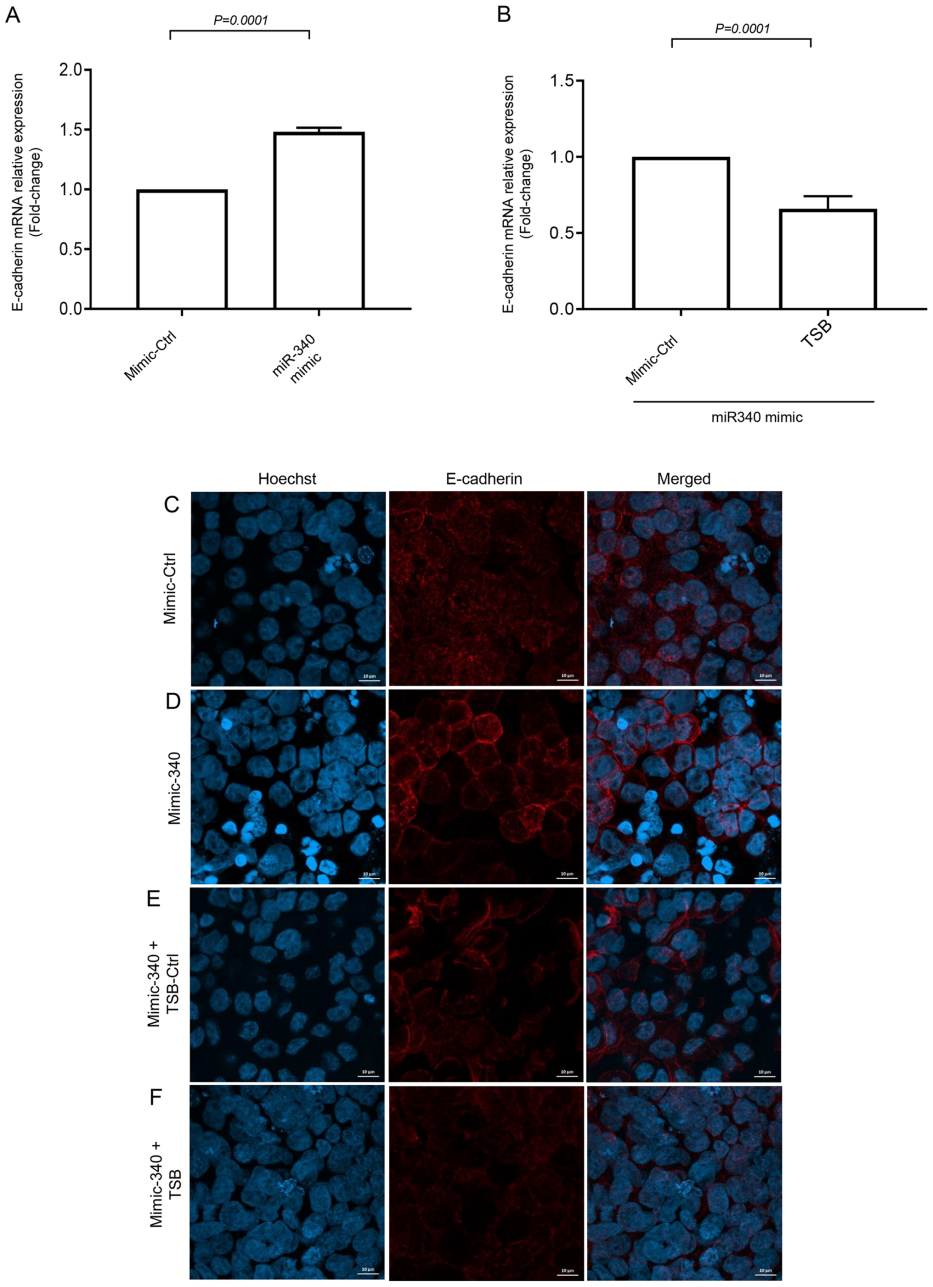
miR-340-5p upregulates E-cad expression in colon cancer cells. E-cad expression was evaluated using (A and B) RT-qPCR and (C-F) confocal microscopy (scale bars, 10 µm). Cells were transfected with miR-340-5p mimic, mimic control, TSB control and TSB for 24 h in low-serum conditions. Colon cancer cells were then stimulated using 10% FBS for 30 min. Relative expressions of E-cad mRNA were quantified using RT-qPCR where β-actin was used as an internal control. Expressions were determined using 2^−ΔΔCq^ method. Data are presented as the mean ± SEM (n=4). miR, microRNA; E-cad, E-cadherin; RT-qPCR, reverse transcription-quantitative PCR; TSB, target site blocker.

To further examine the downstream target protein of FHL2 we examined E-cad expression, which is known to regulate cancer cell metastasis. Short-term serum stimulation significantly decreased E-cad mRNA expression in HT-29 cells (Fig. S5). The inhibition of serum-induced FHL2 mRNA expression by miR-340-5p mimic was found to increase expression of E-cad mRNA (Fig. 7A). Moreover, co-transfection of miR-340-5p mimic with TSB significantly decreased levels of E-cad mRNA expression (Fig. 7B), suggesting that miR-340-5p inhibits FHL2-E-cad axis. Confocal microscopy revealed that continuous distribution of E-cad in the cell-cell junction was attenuated by short-serum stimulation in the stressed cancer cells and miR-340-5p mimic treatment prevented the disruption of E-cad distribution in cellular junction (Fig. 7C and D). As expected, co-transfection of TSB with miR-340-5p mimic, reversed the effect of miR-340-5p mimic in serum-stimulated cells (Fig. 7E and F).

## Discussion

Metastasis of colon cancer is a major cause of colon cancer-related deaths all over the world. Accumulating data suggest that FHL2 oncogene plays an important role in cancer cells migration and invasion. However, the role of FHL2 in colon cancer migration and invasion has not been examined. Herein, we show for the first time that FHL2 acts as an important regulator in colon cancer cell migration and invasion under specific conditions. Furthermore, we found that miR-340-5p has a functional binding site on FHL2 mRNA and that targeting of FHL2 with miR-340-5p mimic reduces cancer cell migration and invasion. Thus, targeting of FHL2 by miR-340-5p could be a useful strategy to inhibit colon cancer metastasis.

FHL2 has been demonstrated to play important roles in the cancer process including proliferation, differentiation and migration (35). However, the literature on FHL2 expression in different cancer types is somewhat complicated and partly contradictory (8,10,12–14). Herein, we first analyzed two colon cancer related microarray datasets and found that FHL2 expression was down-regulated in primary colon cancer compared to matched normal colonic mucosa. This finding is in contrast to two previous studies reporting that FHL2 expression is increased in colon cancer cells (8,36), which could be related to differences in detection methodology. We used two microarray based large datasets to compare FHL2 expression between colon cancer and normal colon tissue while the other studies, reporting higher expression of FHL2 in colon cancer, used western blot and immunohistochemistry. Knowing that FHL2 expression is different in different cancer types, we decided to analyze one large dataset (GSE103512) containing four different cancer types. Our analysis revealed that colon and prostate cancer had similar and significantly higher levels of FHL2 expression compared to breast and non-small cell lung cancer. In addition, we compared primary colon cancer tissue with metastatic colon cancer tissue but observed no significant differences in terms of FHL2 expression, raising a question whether FHL2 plays any role in the pathogenesis of colon cancer. Analysis of GSE97023 microarray dataset revealed that commonly used colon cancer cell lines have two different levels of FHL2 expression, indicating that colon cancer might have two different levels of FHL2 expression depending on the colon cell lines. This observation is in line with Amann *et al* (37) reporting that HT-29 cell line has lower levels of FHL2 expression compared to SW48, CaCo, Lovo, SW480 and HCT116 colon cancer cell lines. We utilized two different cells lines (HT-29 and AZ-97) representing low and relatively high FHL2 expressing colon cancer cells for our subsequent stress related study. To study stress-induced changes in HT-29 and AZ-97 colon cancer cell lines, we cultured both cell lines in low-serum (stress) and serum-rich condition and evaluated FHL2 mRNA expression. Surprisingly, we found that serum condition provoked FHL2 expression in both colon cancer cell lines. Although there was no significant difference in FHL2 expression between primary colon cancer and metastatic colon cancer (microarray data analysis), our observations herein suggest that sudden access to nutrients in stressed cancer cells might increase FHL2 expression and mediate colon cancer pathogenesis.

Accumulating data suggest that miRNAs have characteristic expression patterns in different types of cancers and can regulate cancer progression and metastasis (7,38–41). For example, two recent studies reported that colon cancer tissue has significantly lower levels of miR-340-5p compared to normal colon tissue which is correlated to increased incidence of liver (40) and lymph node (42) metastasis. In this context, we have previously demonstrated that miR-340-5p can reduce colon cancer cell migration by controlling RhoA activity (41), indicating that miR-340-5p can be used to regulate colon cancer pathogenesis. In the present study, we found that transfection of colon cancer cells with miR-340-5p mimic reduced colon cancer cell migration and invasion via FHL2. FHL2 is also shown to regulate cytoskeleton changes in cancer cells. We anticipate that this finding is in line with other studies showing that increased levels of miR-340-5p in breast (43), cervical (44), ovarian (7) and lung (45) cancer reduces metastasis.

To investigate the transient effect of serum on the stressed cancer cells, we cultured transfected cells in low serum conditions for 24 h and then exposed the cells to serum for 30 min. We observed that this short serum exposure increased FHL2 expression in colon cancer cells and that transfection of cells with miR-340-5p mimic significantly reduced serum-induced FHL2 expression both in mRNA and protein levels. Moreover, we found that transfection of HT-29 cells with miR-340-5p reduced cancer cell proliferation in terms of ki67 positive cells. Considering the fact that FHL2 regulates cancer cell migration and proliferation (7,46), these findings indicate that miR-340-5p regulates colon cancer progression with an inverse relationship to FHL2 expression and cell proliferation. Next, we examined whether FHL2 had any binding site for miR-340-5p and bioinformatics based analysis revealed that miR-340-5p had a binding site at 3′-UTR for FHL2 mRNA. We next confirmed the functionality of the binding site using a TSB that specifically competes with miR-340-5p. We observed that exogenous delivery of miR-340-5p into HT-29 cells reduced serum induced cancer cell migration and invasion. The specific TSB also reversed the miR-340-5p mimic-induced FHL2 expression in colon cancer cells, suggesting that the binding site for miR-340-5p on FHL2 mRNA is a functional binding site and it regulates the process of colon cancer cells migration and invasion. It should be noted that FHL2 expression is documented to promote colon cancer cells invasiveness by transforming epithelial cells to mesenchymal cells (8). Moreover, suppression of FHL2 has been shown to down-regulate multiple oncogenes in gastrointestinal cancer cells (36). Whether the suppression of FHL2 by miR-340-5p in colon cancer also have the ability to inhibit other oncogenes remains to be demonstrated.

Knowing that down-regulation of E-cad is an important step in cancer cell progression and metastasis (20,47); we next investigated E-cad expression in miR-340-5p mimic-transfected HT-29 cells 30 min after exposure to serum. We observed that short serum-induced down-regulation or disruption of E-cad in cancer cells was reversed by miR-340-5p and TSB neutralized the effect of miR-340-5p. This finding indicates that inhibition of colon cancer cell migration and invasion by miR-340-5p might be via regulation of E-cad expression. Our finding is in line with a study by Bai *et al* reporting that irregular distribution of E-cad is associated with EMT (48). In addition, Zhang *et al* (21) showed that FHL2 inversely regulates E-cad transcription via a transcription repressor. In this context, it should be noted that down-regulation of E-cad is associated with colon (49), breast (50) and prostate cancer metastasis (51). Notably, our finding that FHL2-E-cad axis plays an important role in colon cancer cell migration and invasion, does not exclude the possibility that other mechanisms might operate in parallel.

Taken together, we conclude that expression of FHL2 in stressed cancer cells play an important role in colon cancer cell migration and invasion. This study shows that inhibition of FHL2 expression by using miR-340-5p mimic reduces colon cancer cells migration and invasion. Moreover, inhibition of FHL2 attenuates cancer cell proliferation and increases E-cad expression in colon cancer cells, suggesting that targeting FHL2 by miR-340-5p might be a useful approach to antagonize colon cancer cell metastasis.

## Supplementary Material

Supporting Data

## Data Availability

The datasets analyzed during the current study are available in the Gene Expression Omnibus database of the National Center for Biotechnology Information (https://www.ncbi.nlm.nih.gov/sites/GDSbrowser?acc=GDS4382, https://www.ncbi.nlm.nih.gov/geo/query/acc.cgi?acc=GSE115313, https://www.ncbi.nlm.nih.gov/sites/GDSbrowser?acc=GDS4393 and http://www.ncbi.nlm.nih.gov/sites/GDSbrowser?acc=GDS4516).

## Abbreviations

FHL2: four and a half LIM domains protein 2
miRNA: microRNAs
E-cad: E-Cadherin
LNA: locked nucleic acids
TSB: target site blocker
