# Supplementary material for: Targeting FHL2-E-cadherin axis by miR-340-5p attenuates colon cancer cell migration and invasion
Source: Oncol Lett. 2021 Jul 3;22(2):637. doi: 10.3892/ol.2021.12898 (PMC8273858; doi:10.3892/ol.2021.12898)

Figure S1. FHL2 expression in colon cancer cell lines. (A) Expression of FHL2 in different types of colon cancer cell lines. FHL2 expression was analyzed in the GSE97023 dataset containing 15 colon cancer cells, some of which are widely used (CaCo2, DLD-1, HCC2988, HCT116, HCT15, HT29, LoVo, SW48 and TC71). Dots represent $\log_2$ normalized sample expression values. (B) FHL2 mRNA expression in AZ-97 and HT-29 cells grown in low-serum conditions. The relative expression of FHL2 was demonstrated using reverse transcription-quantitative PCR, where β-actin was used as an internal control. Expressions were determined using $2^{-\Delta\Delta Cq}$ method. Data are presented as the mean ± SEM (n=4). FHL2, four and a half LIM domains protein 2.

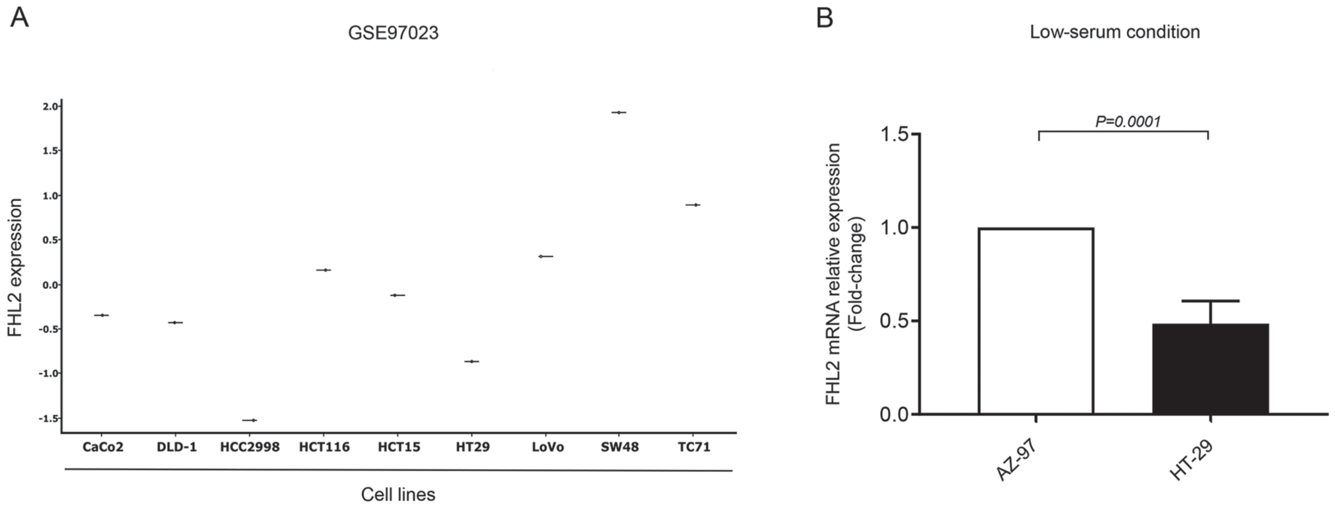

Figure S2. Expression of FHL2 in BC, CRC, NSCLC and PCA in the GSE103512 dataset. The GSE103512 dataset contained 65 BC samples, 57 CRC samples, 60 NSCLC samples and 60 PCA samples. The boxes represent the mean (25-75 percentile) and the whiskers extend from the minimum to the maximum levels and dots represent log_2 normalized sample expression values. FHL2, four and a half LIM domains protein 2; BC, breast cancer; CRC, colorectal cancer; NSCLC, non-small cell lung cancer; PCA, prostate cancer.

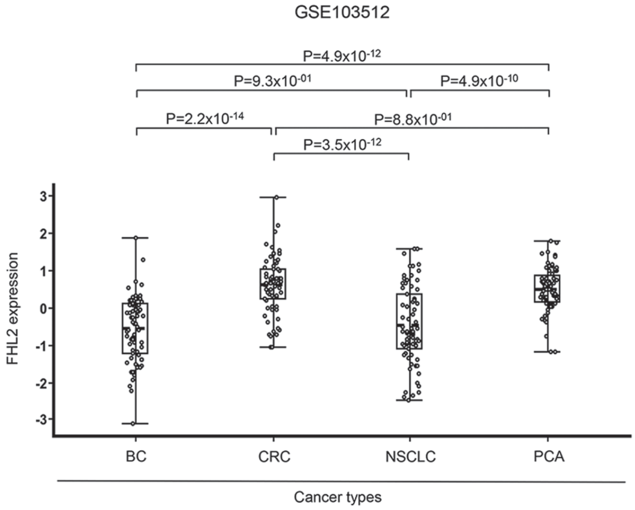

Figure S3. Representative images of the migration assay in Fig. 6A (magnification, x20). Migration of colorectal cancer cells (HT-29) was stimulated by 10% FBS. Cells were transfected with the miR-340-5p mimic, mimic control, TSB control and TSB. Cells were counted microscopically using high power fields in five different fields. miR, microRNA; TSB, target site blocker; Ctrl, control.

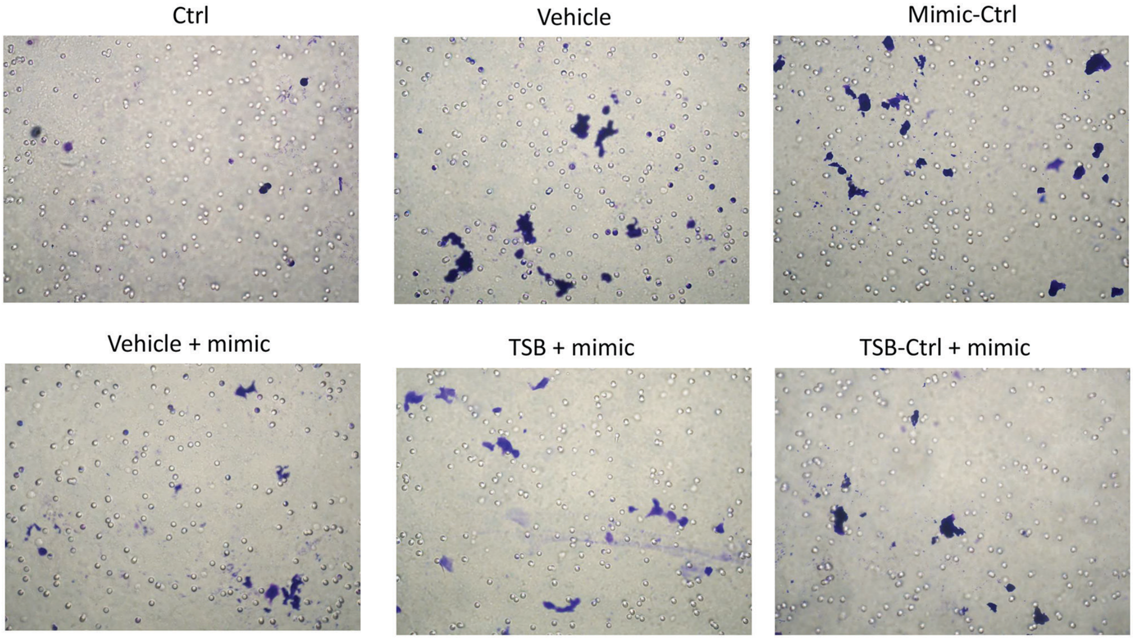

Figure S4. Representative images of the invasion assay in Fig. 6B (magnification, x20). Invasion of colorectal cancer cells (HT-29) through extracellular matrix gel were stimulated by 10% FBS. Cells were transfected with miR-340-5p mimic, mimic control, TSB control and TSB. Cells were counted microscopically using high power fields in five different fields. TSB, target site blocker; Ctrl, control.

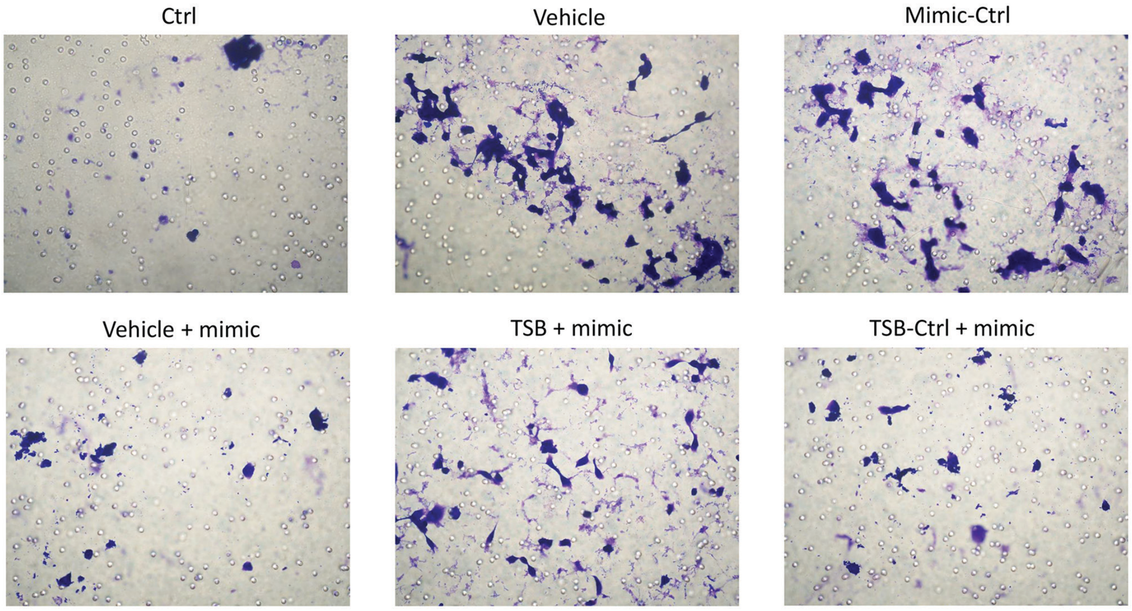

Figure S5. E-cadherin mRNA in HT-29 cells grown in low-serum and serum conditions. E-cadherin relative expression was demonstrated using reverse transcription-quantitative PCR where β-actin was used as an internal control. Expressions were determined using $2^{-\Delta\Delta Cq}$ method. Data are presented as the mean ± SEM (n=4).

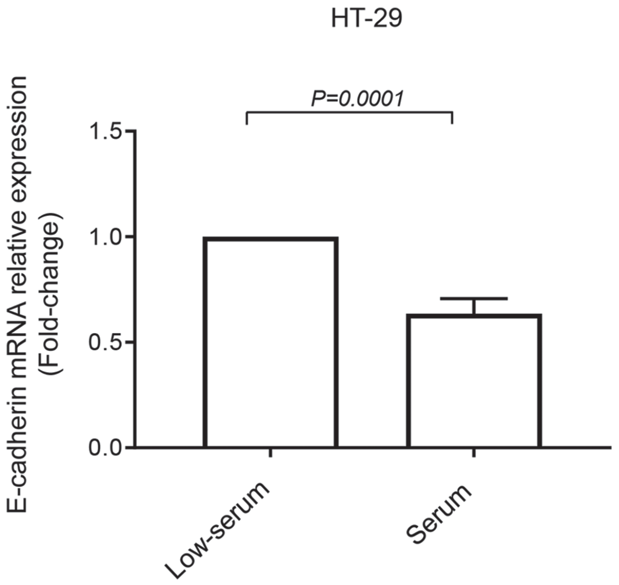

Supplement: Supporting Data [file Supplementary_Data.pdf]
